# Macaúba (*Acrocomia aculeata*) cake from biodiesel processing: a low-cost substrate to produce lipases from *Moniliella spathulata* R25L270 with potential application in the oleochemical industry

**DOI:** 10.1186/s12934-015-0266-9

**Published:** 2015-06-16

**Authors:** Lívia T A Souza, Jamil S Oliveira, Marina Q R B Rodrigues, Vera L dos Santos, Benevides C Pessela, Rodrigo R Resende

**Affiliations:** Departamento de Bioquímica e Imunologia, Universidade Federal de Minas Gerais, Av Antônio Carlos 6627, Pampulha, Caixa Postal 486, Belo Horizonte, MG 31270-901 Brazil; Departamento de Microbiologia, Universidade Federal de Minas Gerais, Av Antônio Carlos 6627, Caixa Postal 486, Belo Horizonte, MG 31270-901 Brazil; Departamento de Biotecnología y Microbiología de Alimentos, Instituto de Investigación en Ciencias de la Alimentación CIAL (CSIC-UAM), Campus de la Universidad Autónoma de Madrid, Nicolás Cabrera 9, 28049 Madrid, Spain; Instituto Nanocell and Departamento de Bioquímica e Imunologia, Instituto de Ciências Biológicas, Universidade Federal de Minas Gerais, Av Antônio Carlos 6627, Pampulha, Caixa Postal 486, Belo Horizonte, MG 31270-901 Brazil

**Keywords:** Macaúba cake, Lipases, Yeast, *Moniliella spathulata*, Submerged fermentation, Hydrolysis, Oils, Biodiesel

## Abstract

**Background:**

Biodiesel industry wastes were evaluated as supplements for lipase production by *Moniliella spathulata* R25L270, which is newly identified yeast with great lipolytic potential. Macaúba cake (MC), used for the first time in this work as inducer to produce lipases, and residual oil (RO) were mixed to maximise enzyme production. The lipase secreted was biochemically characterised.

**Results:**

The best ratio for the mixture (MC:RO) was 0.66:0.34 and the fitted values for lipase activity and total protein concentration were 0.98 U mL^−1^ and 0.356 mg mL^−1^, respectively. Maximum activity obtained (2.47 U mL^−1^) was achieved at 31.5°C and pH 6.7, and the enzyme was stable in this condition. A novel enzyme was purified and identified for the first time by mass spectrometry. The lipase efficiently hydrolysed different natural oils and exhibited selectivity in the production of eicosapentaenoic acid from fish oil.

**Conclusion:**

The use of MC and RO as a supplement to produce the new lipase from *M. spathulata* R25L270 may be one alternative for reducing lipase production costs and simultaneously adding value to biodiesel industry residues. The potential application of the lipase in the oleochemical industry was demonstrated by its pH and temperature stabilities and selective hydrolysis.

## Background

The increase in global biodiesel demand is evident; however, the following two main challenges still reduce the economic viability of this renewable fuel: (a) the feedstock cost and (b) the high volume of glycerol and residues generated by the production process [1, 2]. One way to eliminate these wastes is to provide added value based on their utilization to produce biotechnological products such as enzymes and other bio products. In this context, the use of lipid-rich waste as substrate for the production of microbial lipases has been demonstrated as a viable technique to reduce the enzyme cost while solving the pollution problems caused by the disposal of these residues [3]. In this work, three residues from biodiesel processing (macaúba cake, residual oil and residual water) were evaluated with respect to their potential use in lipase production by *Moniliella spathulata* R25L270. Biodiesel processing of the Macaúba palm (*Acrocomia aculeata*) was chosen due to its great use in Brazilian industry to biodiesel production as a feedstock with the potential to produce 1,500–5,000 kg oil ha^−1^, which is only lower to the production of palm oil (*Elaeis guineensis*) [4]. The use of Macaúba oil in Brazil is escalating at a remarkable rate to increase the commercial competitiveness of biofuel and support development in the producing areas [5].

Lipases (EC. 3.1.1.3) are able to modify triglycerides and other esters at the water–oil interface by catalysing hydrolysis and synthesis reactions [6]. Because of their versatility and specificity, lipases are promising enzymes for use in the oleochemical industry. The world’s consumption of fatty acids and fatty alcohols in 2010 was 6 and 2.5 million tons, respectively, and is expected to reach 7.5 and 3.5 million tons in 2020 [7]. Furthermore, lipases are sold in large volumes for use in other industrial applications, for example, in detergent formulation, food processing, biodiesel production, racemic resolution, pharmaceuticals, and waste treatment [6].

Yeast lipases have a great importance in the enzyme market due their solubility, temperature and pH stabilities. *Candida*, *Rhodotorula*, *Debaryomyces*, *Trischosporon* and *Yarrowia* are the main genera with species that produce lipases [8]. The strain used in this work, *M. spathulata* R25L270, was isolated from Brazilian butter cheese (*Requeijão do Norte*), and its potential lipolytic was recently revealed (date submitted). However, there is not description or identification of the lipases it produces. Hence, the aims of this study were to evaluate and maximise lipase production in low-cost culture medium using biodiesel process residues followed by biochemical characterisation, purification, identification and application of the produced lipases in catalysis of the hydrolysis of vegetal and fish oils.

## Methods

### Microorganism and inoculum

The strain *M. spathulata* R25L270 from the culture collection of the Universidade Federal de Minas Gerais (Brazil) was isolated from Brazilian butter cheese (*Requeijão do Norte*), and its lipolytic potential has been recently identified (submitted for publication). Cultures of this yeast were maintained in GYMP broth (glucose 20 g L^−1^, yeast extract 5 g L^−1^, malt extract 10 g L^−1^, NaH_2_PO_4_ 2 g L^−1^ and glycerol 20 l L^−1^) at −80°C. For inoculum preparation, fresh cells of the yeast cultivated on Sabouraud agar medium composed of glucose 20 g L^−1^, peptone 10 g L^−1^ and yeast extract 5 g L^−1^ were used. Cells were grown with shaking at 30°C (200 rpm) for 24 h. The cells were then recovered by centrifugation (5,000×*g*, 10 min) and suspended in the fermentation medium at the desired initial culture density (DO_600_ = 0.1).

### Experimental procedure for lipase production using biodiesel wastes

Fermentation assays to evaluate the possibility of using waste from biodiesel industry as a supplement for lipase production were performed in three consecutive stages. First, the influence of nitrogen sources (20 g L^−1^) such as peptone G, water peptone, bacteriological peptone, proteose peptone, bacto-tryptone and peptone A on lipase activity was evaluated. These assays were performed in 250 mL Erlenmeyer flasks containing 50 mL of medium composed of NH_4_NO_3_ 1 g L^−1^; KH_2_PO_4_ 1 g L^−1^; MgSO_4_·7H_2_O 5 g L^−1^; and 10 g L^−1^ of olive oil at pH 6.5. In the second step, the medium containing the best nitrogen source (peptone A) was supplemented with macaúba cake obtained from fruit pulp (40 g L^−1^), oil residual extracted from diatomaceous earth (10 g L^−1^) or liquid effluent collected from washing biodiesel tanks (500 l L^−1^) to evaluate the potential use of these oil sources for lipase production. In the last stage, a simplex lattice mixture design was used to study the effects of binary blends of macaúba cake and residual oil (MC:RO) on lipase secretion in terms of enzyme activity and concentration. The MC:RO proportions study and the respective observed and adjusted responses obtained: For MC, the maximal concentration assayed (100:0) was 40 g L^−1^, and for RO the maximal concentration assayed (0:100) was 10 g L^−1^. In all cases, after inoculation at the initial optical density (DO_600_ = 0.1), the flasks were incubated in a rotary shaker at 30°C and 200 rpm for 120 h. The cells were then recovered by centrifugation (8,000×*g*, 10 min), and their supernatants were used for the determination of lipase activity. Regression models were adjusted to explain the influence of the MC:RO proportion on the extracellular lipase activity and total protein concentration in the supernatant. The adjusted models were then used to estimate the optimal proportion for the two response variables using a desirability function. Statistical analysis and graphing were performed with MINITAB 16 (Stat-Ease, Inc., Minneapolis, MN, USA) using a 95% confidence cut-off (α = 0.05).

### Lipase activity assay

Activity was continuously followed spectrophotometrically by the increase in the absorbance at 410 nm caused by the hydrolysis of *p*-nitrophenyl palmitate (*p*-NPP) using a Varioskan microplate reader [9, 10]. One unit of lipase (U) was defined as the amount of enzyme that releases 1 µmol *p*-nitrophenol (*p*NP) per min in the assay conditions (pH 8.0, 37°C).

### Biochemical characterisation of *M. spathulata* R25L270 lipase

All tests were conducted in triplicate using the crude supernatant obtained during cultivation of the yeast in medium supplemented with olive oil.

#### Storage stability

Crude lipase storage stability was evaluated by storing the cell-free culture supernatant at the following temperatures: freezer (−20°C), ultra-freezer (−80°C) and refrigerator (4°C). Lipase activity was measured every 7 days using the lipase activity assay described above.

#### Determination of pH and temperature optimum of lipase

A central composite design (2^2^ + 2 × 2 + 5) was employed to study the influence of reaction pH and temperature on *M. spathulata* R25L270 lipase activity. The experimental matrix assayed is presented in Table 1. Each parameter combination was repeated at least twice. The lipase activity response (U mL^−1^) was measured using the lipase activity assay described above. The experimental design and regression analysis were performed with MINITAB 16 software, and the response surface plot was generated by Sigma Plot 10.0 software.

**Table 1:** Central composite design matrix used to study the influence of reactional temperature (°C) and pH on lipase activity (U mL^−1^) and the respective observed (y) and fitted ($\hat{y}$) values and residual errors (ε)

Central composite design		Lipase activity (U mL^−1^)		
Temperature (°C)	pH	y	$\hat{y}$	ε
45	6.5	2.44	2.17	0.271
45	6.5	2.48	2.17	0.319
45	6.5	2.32	2.17	0.151
45	9.5	0.88	1.08	−0.200
45	9.5	0.88	1.08	−0.196
45	9.5	0.86	1.08	−0.219
45	8	2.09	1.94	0.147
45	8	1.90	1.94	−0.042
45	8	1.88	1.94	−0.056
45	8	1.93	1.94	−0.006
45	8	1.90	1.94	−0.042
65	8	0.48	0.39	0.080
65	8	0.49	0.39	0.091
25	8	2.15	2.17	−0.015
25	8	2.14	2.17	−0.031
30.8	6.94	2.30	2.46	−0.161
30.8	6.94	2.31	2.46	−0.155
30.8	6.94	2.41	2.46	−0.052
30.8	9.06	1.75	1.70	0.054
30.8	9.06	1.78	1.70	0.079
30.8	9.06	1.96	1.70	0.262
59.1	6.94	0.89	1.21	−0.324
59.1	6.94	0.82	1.21	−0.393
59.1	9.06	0.65	0.45	0.200
59.1	9.06	0.68	0.45	0.236

#### Thermostability of *M. spathulata* R25L270 lipase

The thermostability of the enzyme was studied at different pH and temperature conditions. The crude lipase supernatant was diluted 1:1 (v/v) in 50 mM Tris–HCl buffers pH (6.5, 7.5 and 8.5) and incubated at 45 and 50°C, respectively. Samples were taken at determined interval times, and the enzyme activity was immediately measured using the standard assay. The residual activity was calculated by taking the enzyme activity at 0 min incubation as 100%.

### One-step purification/immobilization of lipases from *M. spathulata* R25L270 on phenyl-Sepharose

Phenyl-Sepharose (1 g) was suspended in 8 mL of crude supernatant culture containing approximately 0.8 U mL^−1^ of lipases and 2 mg of total protein and 2 mL of phosphate buffer (5 mM, pH 7). The suspensions were gently stirred at room temperature. At 10-min intervals, samples of the suspensions were withdrawn and centrifuged (8,000×*g*; 3 min) to obtain the supernatants, and their enzyme activity was analysed as described above (“Lipase activity assay”). After immobilization, the suspension was filtered in a sintered glass filter and vacuum dried. Proteins were desorbed by suspending the immobilized enzyme in a 1:10 (w/v) ratio of 25 mM sodium phosphate at pH 7.0 and 25°C containing different concentrations [0.02% until 0.1% (v/v)] of Triton X-100 until full desorption [11].

### SDS-PAGE analysis

The protein profile of the culture supernatant was determined by SDS-PAGE using a Laemmli system with 12.5% acrylamide [12]. The proteins were stained with Coomassie blue dye [13] and Schiff reagent [14]. A native PAGE with 10% acrylamide was also done for it the gel was prepared without SDS and the samples were not boiled before loading in the gel. The lipase activity in the gel was detected using tributyrin as the substrate according to [15].

### Protein identification by mass spectrometry (MS)

The profile of protein desorbed with a gradient of Triton X-100 was analysed by SDS-PAGE. The observed bands were analysed by MS at the Centro de Biología Molecular Severo Ochoa, CBM-CSIC campus Universidad Autónoma de Madrid, Plataforma in Red de Proteómica Carlos III. After drying, gel bands or spots were destained in acetonitrile:water (ACN:H_2_O, 1:1) and digested in situ with sequencing-grade trypsin (Promega, Madison, WI), according to Shevchenko et al. [16]. The desalted protein digest was dried, resuspended in 10 µL 0.1% formic acid and analysed by RP-LC–MS/MS in an Easy-nLC II system coupled to an ion trap LTQ-Orbitrap-Velos-Pro mass spectrometer (Thermo Scientific). The peptides were concentrated (on-line) by reverse phase chromatography using a 0.1 mm × 20 mm C18 RP precolumn (Acclaim PepMap® 100, nanoViper, Thermo Scientific) and then separated using a 0.075 mm × 250 mm C18 RP column (Acclaim PepMap® 100, nanoViper, Thermo Scientific) operating at 0.3 μL min^−1^. Peptides were eluted using a 90-min gradient of 5–40% solvent (solvent A: 0.1% formic acid in water, solvent B: 0.1% formic acid, 80% acetonitrile in water). ESI ionisation was performed using a Nano-bore emitter Stainless Steel ID 30 μm (Proxeon) interface. The Orbitrap resolution was set at 30,000. Peptides were detected in survey scans from 400 to 1,600 amu (1 μscan), followed by 15 data-dependent MS/MS scans (Top 15) using an isolation width of 2 u (in mass-to-charge ratio units), normalised collision energy of 35%, and dynamic exclusion applied during 30-s periods. Peptide identification from raw data was carried out using the SEQUEST HT algorithm (Proteome Discoverer 1.4, Thermo Scientific). A database search was performed against uniprot-fungi.fasta. The following constraints were used for the searches: tryptic cleavage after Arg and Lys, up to two missed cleavage sites, and tolerances of 20 ppm for precursor ions and 0.8 Da for MS/MS fragment ions. The searches were performed allowing optional Met oxidation and Cys carbamidomethylation. The search was performed against a decoy database (integrated decoy approach) using a false discovery rate (FDR) <0.01.

### Hydrolysis of vegetal and fish oil

The hydrolytic activity of lipase from *M. spathulata* R25L270 was determined for emulsified vegetable oils (corn, sunflower, soybean, olive, canola, pequi, almond, macaúba and sesame) according to Soares et al. [17]. The fatty acids formed were titrated with 20 mM sodium hydroxide solution in the presence of phenolphthalein as an indicator. One international unit (U) of activity was defined as the amount of enzyme that releases 1 μmol free fatty acid per minute under the assay conditions. Fish oil hydrolysis was performed in presence of cyclohexane, as proposed by Fernández-Lorente et al. [18]. The concentration of free fatty acids in the organic phase was determined by RP-HPLC (Spectra Physic SP 100 coupled with a UV detector SpectraPhysic SP 8450) using a reversed-phase column (Ultrabase C18, 4.6 mm i.d. × 150 mm, 5 μm particle) [18].

## Results and discussion

### Production of lipases by *M. spathulata* R25L270 grown on biodiesel waste

The primary objective of this work was to analyse the suitability of biodiesel residues as a substrate for the growth of *M. spathulata* R25L270 and lipase production. Lipase production was studied using the following consecutive analyses: (a) effect of organic nitrogen source, (b) effect of individual biodiesel residues and (c) mixture of biodiesel residues. Nitrogen sources play a crucial role in regulating the synthesis of hydrolases, and the production of fungal lipases appears to require a high concentration of nitrogen compared to the production of other enzymes [19]. As shown in Table 2, among the organic nitrogen sources tested in this work, peptone A and bacto-tryptone exhibited the highest lipase activities (0.69 ± 0.03 and 0.63 ± 0.07 U mL^−1^, respectively) and supported good cell growth (25.0 and 28.2 OD nm, respectively). In contrast, water peptone (0.03 ± 0.0 U mL^−1^) and peptone G (0.0 U mL^−1^) showed inhibition of lipase activity compared to bacteriological peptone (0.34 ± 0.4 U mL^−1^), the most commonly used peptone. The nitrogen sources differed in their nutritive value, degree of peptide hydrolysis and amino acid content; these factors are associated with the specific nutritional requirements of yeast and can explain the influence on growth and lipase production. For *Yarrowia lipolytica*, the highest level of lipase activity was obtained in the presence of casein hydrolysate (tryptone N_1_), and formulation media containing casamino acids led to low lipolytic productivity [20]. *Candida viswanathii* presented high cell growth and lipase production when cultivated in Vogel’s medium supplemented with peptone, tryptone and yeast extract. However, the use of urea, NH_4_Cl and (NH_4_)_2_SO_4_ inhibited lipase secretion but supported cell growth [21].

**Table 2:** Effect of organic nitrogen source on lipase secretion and growth of *M. spathulata* R25L270

Nitrogen source	Lipase activity (U mL^−1^)	Growth OD_600 nm_
Peptone G	0 ± 0	9.4
Water peptone	0.03 ± 0.0	25.6
Peptone bacteriological	0.34 ± 0.03	23.8
Proteose peptone	0.46 ± 0.02	14.4
Bacto-typtone	0.63 ± 0.07	28.2
Peptone A	0.69 ± 0.03	25.0

After selecting the most suitable organic nitrogen source for the secretion of lipases by *M. spathulata* R25L270 cultivated in medium with olive oil as an inducer, the basal liquid medium was supplemented individually with three different residues from the macaúba biodiesel production process, macaúba cake, residual oil, and liquid waste. The lipase activity and yeast growth were monitored. There was no growth of the yeast strain in medium supplemented with liquid waste. The microbial growth inhibition caused by this substrate may be associated with the addition of potassium hydroxide required to elevate the pH of these residues from 1.5 to 6.5 or the presence of contaminants and trace solvents. The low pH of the effluent is associate with the addition of acid required to neutralization and washing biodiesel step [22]. When the yeast was cultivated in medium enriched with residual oil instead of olive oil, the extracellular lipase activity was 0.37 ± 0.08 U mL^−1^, but good growth of the yeast was not observed. According Monteiro et al. [23] the composition of this residual oil indicated the presence of hexadecanoic acid (22.92%), 9,12-octadecadienoic acid (36.74%), 9-octadecenoic acid (34.74%), and octadecanoic acid (5.60%) [23]. Lipase activity was better when the medium was supplemented with olive oil (0.69 U mL^−1^) as compared with the medium supplemented only with residual oil (0.37 U mL^−1^), probably due the lower content of 9-octadecenoic acid. Certain oil residues, such as grease wastes, inhibit microorganism growth and/or lipase secretion because of this different agro-waste residues, such as wheat bran, rice bran, and oil cake, are used in combination [24]. The maximum lipolytic activity (0.7 ± 0.04 U mL^−1^) was achieved when the yeast was grown in a macaúba cake-supplemented medium. In this fermentation condition, it was not possible to measure the optical density of the culture due to the presence of suspended solids and turbidity, so we cannot link maximal lipolytic activity and growth.

To study the influence of the macaúba cake (MC) and residual oil (RO) mixture on lipase secretion by *M. spathulata* R25L270, a simplex centroid mixture design was used. The levels of the two factors were selected based on preliminary studies carried out in our laboratory. The experimental design and the results of the mixture trials are presented in the Table 3. Sequential model fitting of the lipase activity (U mL^−1^) and total protein concentration (mg mL^−1^) showed that the quadratic model is the most appropriate to explain the influence of the MC:RO mixture on lipase secretion by *M. spathulata* R25L270 as shown in Eqs. (1) and (2).

1 $$ {\text{LA }} = \, 0. 7 3 3 {\text{MC }} + \, 0.0 6 5 {\text{RO }} + { 2}. 1 1 3 {\text{ MC}} \cdot {\text{RO}} $$

2 $$ {\text{LP }} = \, 0. 3 2 6 {\text{MC }} + \, 0.0 9 8 {\text{RO }} + \, 0. 4 7 9 {\text{ MC}} \cdot {\text{RO}} $$

where, LA corresponds to lipase activity (U mL^−1^), LP corresponds to total protein (mg mL^−1^), MC corresponds to macaúba cake proportion, and RO corresponds to residual oil proportion.

**Table 3:** Simplex lattice mixture design matrix used to study the influence of macaúba cake and residual oil mixture on lipase secretion by *M. sphatulata* R25L270 presenting the MC:RO proportions and the respective observed (y) and fitted ($\hat{y}$) values and residual errors (ε) for supernatant lipase activity (U mL^−1^) and supernatant total protein (mg mL^−1^) responses

Simplex lattice mixture design		Lipase activity (U mL^−1^)			Total protein concentration (mg mL^−1^)		
MC proportion	RO proportion	y	$\hat{y}$	ε	y	$\hat{y}$	ε
1.00	0.00	0.79	0.73	0.056	0.399	0.326	0.0726
0.00	1.00	0.04	0.07	−0.026	0.098	0.098	0.0003
0.50	0.50	1.02	0.93	0.092	0.318	0.332	−0.0139
0.75	0.25	0.85	0.96	−0.113	0.321	0.359	−0.0381
0.25	0.75	0.69	0.63	0.061	0.248	0.245	0.0032
1.00	0.00	0.77	0.73	0.036	0.273	0.326	−0.0534
0.00	1.00	0.04	0.07	−0.026	0.09	0.098	−0.0077
0.50	0.50	0.96	0.93	0.032	0.381	0.332	0.0491
0.75	0.25	0.85	0.96	−0.113	0.347	0.359	−0.0121

**Figure 1:**
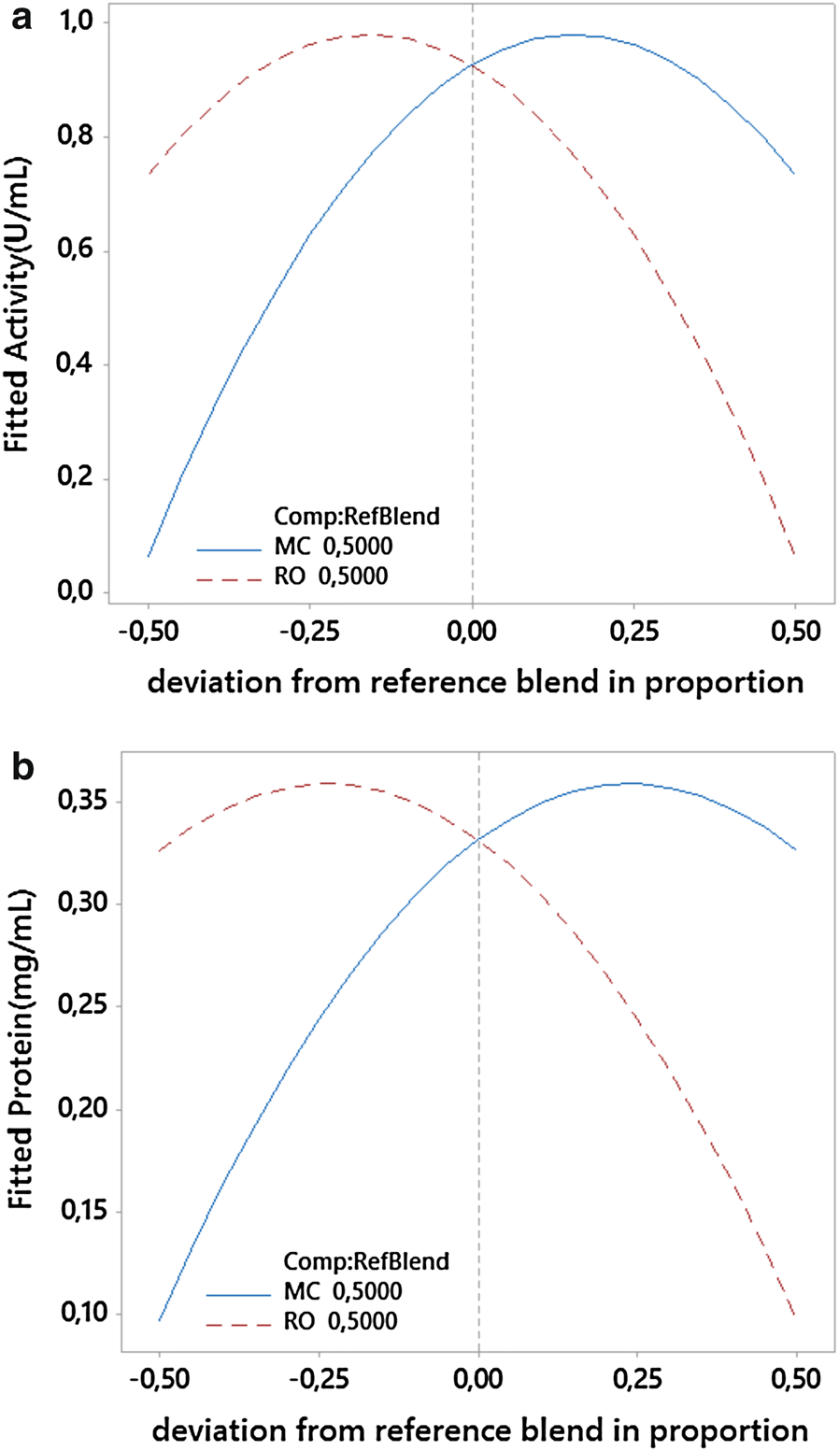
Cox response trace plot for fitted supernatant lipase activity (U mL^−1^) (**a**) and supernatant total protein concentration (mg mL^−1^) (**b**) versus the MC:RO deviation proportions from the reference blend 0.5:0.5 for MC (*solid line*) or RO (*dashed line*).

Analysis of variance (ANOVA) of the quadratic response surface model was used to assess the adequacy of the model and showed that the two adjusted models can significantly (p value = 0.000 and p value = 0.002, for LA and LP, respectively) explain the variation in responses based on the different MO:RO proportions in the medium. The R^2^ values indicated that 95.91 and 87.85% of the observed variation in supernatant lipase activity and total protein concentration, respectively, can be explained by this model. The significant, positive and relatively strong interaction between macaúba cake (MC) and residual oil (RO) observed in both adjusted equations (Eqs. 1, 2) reveal the existence of synergism between the two components of the mixture. The Cox response trace plots for fitted LA and LP are presented in Figure 1. By using a composite desirability tool and the adjusted equations for LA and LP to maximise both responses simultaneously, we found that the best ratio for the mixture is 0.66:0.34 (MC:RO) and the fitted values for LA and LP are 0.98 U mL^−1^ and 0.356 mg mL^−1^, respectively. There is a range (from 0.639 to 0.675 of MC) in which the fitted value for LA is 0.98 U mL^−1^, so we present these values as the best combinations to obtain lipase activity and protein. These novel fermentation conditions can be used instead of olive oil for the induction of lipase secretion by *M. spathulata* R25L270. Additionally, this experimental design can be applied to study the potential use of mixtures of biodiesel residuals as substrates for lipase production by other yeasts.

In general, the microbial pattern of lipase production is associated with cell growth and substrate consumption, and it occurs in stress conditions. At the initial stage, cell-bound lipase (at basal levels) is able to hydrolyse the lipid (inducer) present in the substrate at a reaction rate high enough to start cell growth and enzyme expression [25]. As fermentation continues and substrate availability decreases, the production of extracellular lipases becomes necessary to promote substrate uptake and ensure cell survival [25]. At this time, enzyme release increases the probability of enzyme–substrate contact and nutrient assimilation [25]. This mechanism is generally dependent on cellular regulatory mechanisms elicited by the components of the substrates, mainly lipid inducers.

The yeast *M. spathulata* R25L270 was recently described as a microorganism with lipolytic potential, and its nutritional requirements for lipase secretion are unknown. Oil cakes contain residual nutrients that can serve as both carbon and nitrogen sources. The use of oil cakes such as babassu [26], *Jatropha curcas* [27], soybean [28], mustard [29], sesame [30], coconut [30], and ground nut [30] has been successful used to obtain lipases from different microorganisms.

We report the first use of macaúba cake for lipase production. Macaúba cake is composed of crude protein, fibre and lipids and has an elemental composition of C 50.93%, O 40.68%, H 6.59%, N 1.60% and S 0.197% [31]. This composition i improved lipase secretion and supported biomass growth of *M. spathulata* R25L270.

The mixture of macaúba cake (MC) with residual oil (RO) increased the lipase activity, probably due to the high amount of essential nutrients, mainly from MC, and inducer concentration, mainly from RO. These increases can explain the significant interaction found between the two components of the mixture. Some microorganisms need inducers such as natural oils, fatty acids, fatty esters, sterols, bile salts and Tween to produce and secrete lipases [6]. The lipase activity depends on the inducer type and concentration of the fatty acid constituents of the substrate. Abdelmoez et al. [32] tested olive oil, fatty acid residues, soap stock and a mixture of these three components as inducers to obtain lipases by *Candida rugosa* ATCC 14830. Lipase secretion was dependent on lipid type and concentration, and the maximum activities of the produced lipases utilising olive oil (0.5%), fatty acid residues (1.5%) and soapstock (1%) were found to be 12, 7 and 7.4 U mL^−1^, respectively. Using a mixture of this substrate (the concentration is not mentioned), the lipase activity achieved was 10 U mL^−1^.

### Biochemical characterisation of lipases from *M. spathulata* R25L270

**Figure 2:**
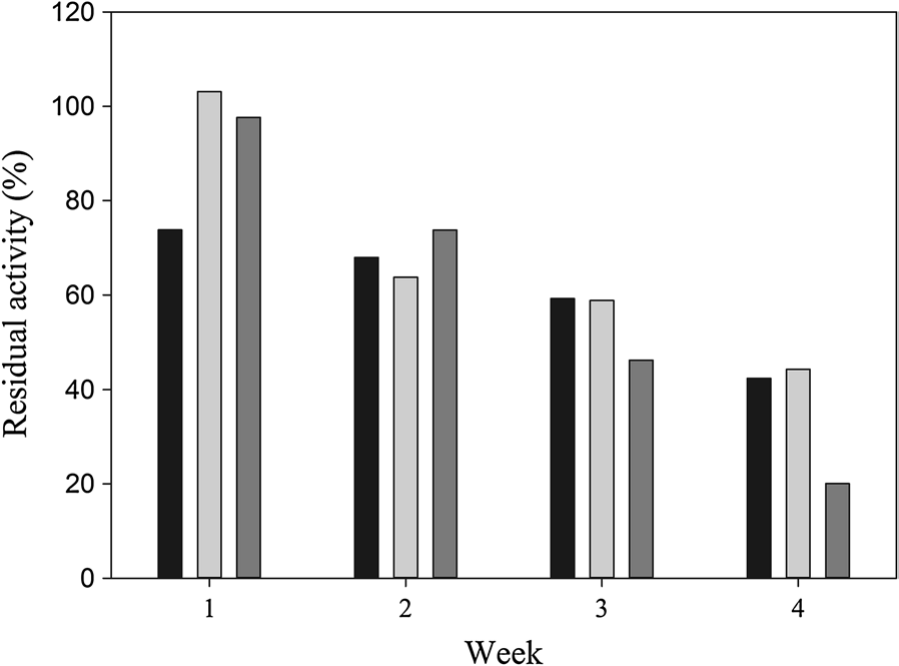
Storage stability of crude lipases from *M. spathulata* R25L270. Freezer (−20°C) (*dark gray*), ultra-freezer (−80°C) (*light gray*) and fridge (4°C) (*black*).

Biochemical characterisation of new enzymes is critical to determining catalytic properties of the protein and their compatibility for use in bioprocesses. One important prerequisite for laboratory-scale tests and future industrial enzyme application is to determine the lipase storage stability. Therefore, the *M. spathulata* R25L270 culture supernatant was stored in a refrigerator (4°C), freezer (−20°C), and ultra-freezer (−80°C), and the enzyme activity was measured weekly for 1 month. As shown in Figure 2, the residual activity was at least 40% after 1 month of storage in a refrigerator (4°C) and ultra-freezer (−80°C). Crude lipases from *Fusarium solani* N4-2 retained almost 70 and 50% of the initial activity when stored for 1 month at 4°C and room temperature, respectively [33]. The crude lipase from *Aspergillus japonicus* retained approximately 80% of its initial activity after 4 weeks of storage at −80°C [9]. As exemplified above, lipase storage stability depends on the enzyme source. The observed storage stability of lipase from *M. spathulata* R25L270 without the addition of stabilising agents indicates its potential use in bioprocesses.

In the present work, a central composite design was used to obtain a response surface model to explain the effect of reaction pH and temperature on lipase activity. Table 1 shows the combinations of the independent variables, reaction pH and temperature, and the respective observed and adjusted values and residual errors obtained for the response lipase activity (LA) (U mL^−1^).

The second-order polynomial model adjusted for lipase activity is given in Eq. 3.

3 $$ {\text{LA}} = - 5. 4 8 9 + 0. 10 4 {\text{T}} + 1. 8 8 6 {\text{P}} - 0.00 2 {\text{T}}^{ 2} - 0. 1 4 1 {\text{P}}^{ 2} $$

where LA corresponds to lipase activity (U mL^−1^), T corresponds to temperature, and P corresponds to pH.

**Figure 3:**
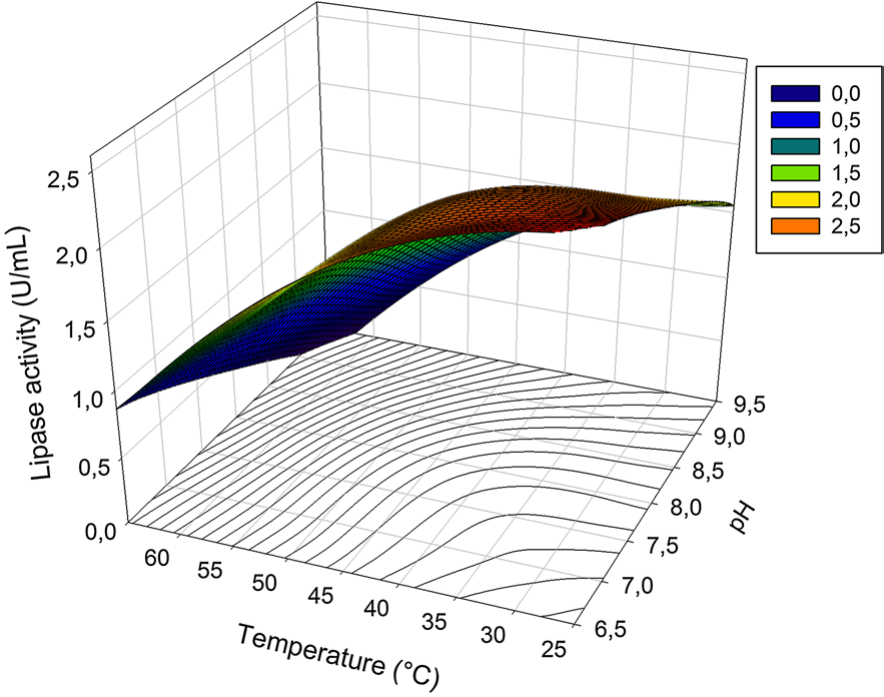
Response surface plot for adjusted lipase activity (U mL^−1^) versus reactional temperature (°C) and pH.

The ANOVA results indicated that the adjusted model is significant (p value = 0.0001), and 92.86% of the observed variation in the lipase activity can be explained by this model as indicated by the R^2^ value. The contour and response surface plots for the adjusted LA are shown in Figure 3. According to the adjusted model for LA, the maximal enzymatic activity (2.47 U mL^−1^) is achieved at 31.5°C and pH 6.7. Additionally, at least 80% of the maximal activity value can be achieved by using different combinations of temperature (25–48°C) and pH (6.5–8.4) ranges. This broad area of maximum performance allows the exploitation of many combinations of temperature and pH, indicating the versatility of this enzyme and potential for application in different bioprocesses. These values are in accordance with those reported by Goldbeck and Filho [34], who studied the lipase from *Metschnikowia pulcherrima* for which the optimal range for temperature and pH was 37–47°C and 6.0–7.4, respectively.

**Figure 4:**
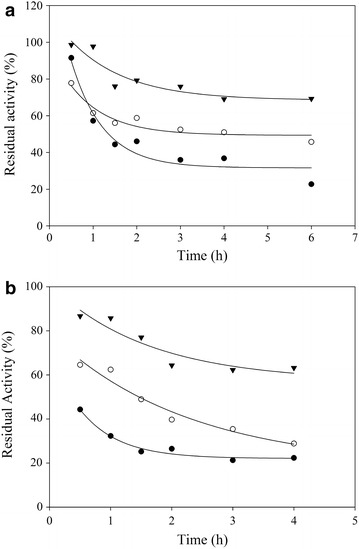
Thermostability of lipase from *M. spathulata* R25L270 was measured by incubating the lipase in 50 mM TRIS–HCl buffer pH 8.5 (*black circle*), pH 7.5 (*white circle*) and pH 6.5 (*triangle*) at 45°C (**a**) and 50°C (**b**). Residual lipase activity (%) was calculated relative to the initial activity.

The thermostability of lipases from *M. spathulata* R25L270 was studied by incubation of crude supernatant in 50 mM Tris–HCl buffer (pH 6.5–7.5–8.5) at 45 and 55°C. As shown in Figure 4, of all the temperatures tested, the enzyme was most stable at pH 6.5. These results are in accordance with those obtained during the study of effect of pH and temperature on lipase activity. As shown in Figure 3, the enzyme activity decreases with increasing in pH and temperature, probably due the low enzyme stability under these conditions. The lipase from *M. spathulata* R25L270 retained 70% of the initial activity after incubation for 4 h at 45°C at pH 6.5. The thermostability of lipases from *M. spathulata* R25L270 was elevated compared to that obtained from other microbial sources such as *Penicillum crustosum* (25% of initial enzymatic activity after incubation for 1 h at 45°C) [28], *Pseudomonas* sp DMVR 46 (28% of initial enzymatic activity after incubation for 4 h at 40°C) [35] and *Pichia lynferdii* Y-7723 (35% of initial enzymatic activity after incubation for 10 min at 50°C) [36].

The process of oil modification catalysed by lipases typically occurs at temperatures close to 40°C, as observed for biodiesel production [5] and aroma synthesis [35]. The moderate temperature is necessary to homogenise the reaction medium due to the high oil viscosity and the existence of solid oil at room temperature. The thermostability observed for the novel lipases studied indicates that the culture supernatant containing the enzyme has the potential to be successfully applied in bioprocesses.

### Purification of lipases from *M. spathulata* R25L270 on phenyl-Sepharose

**Figure 5:**
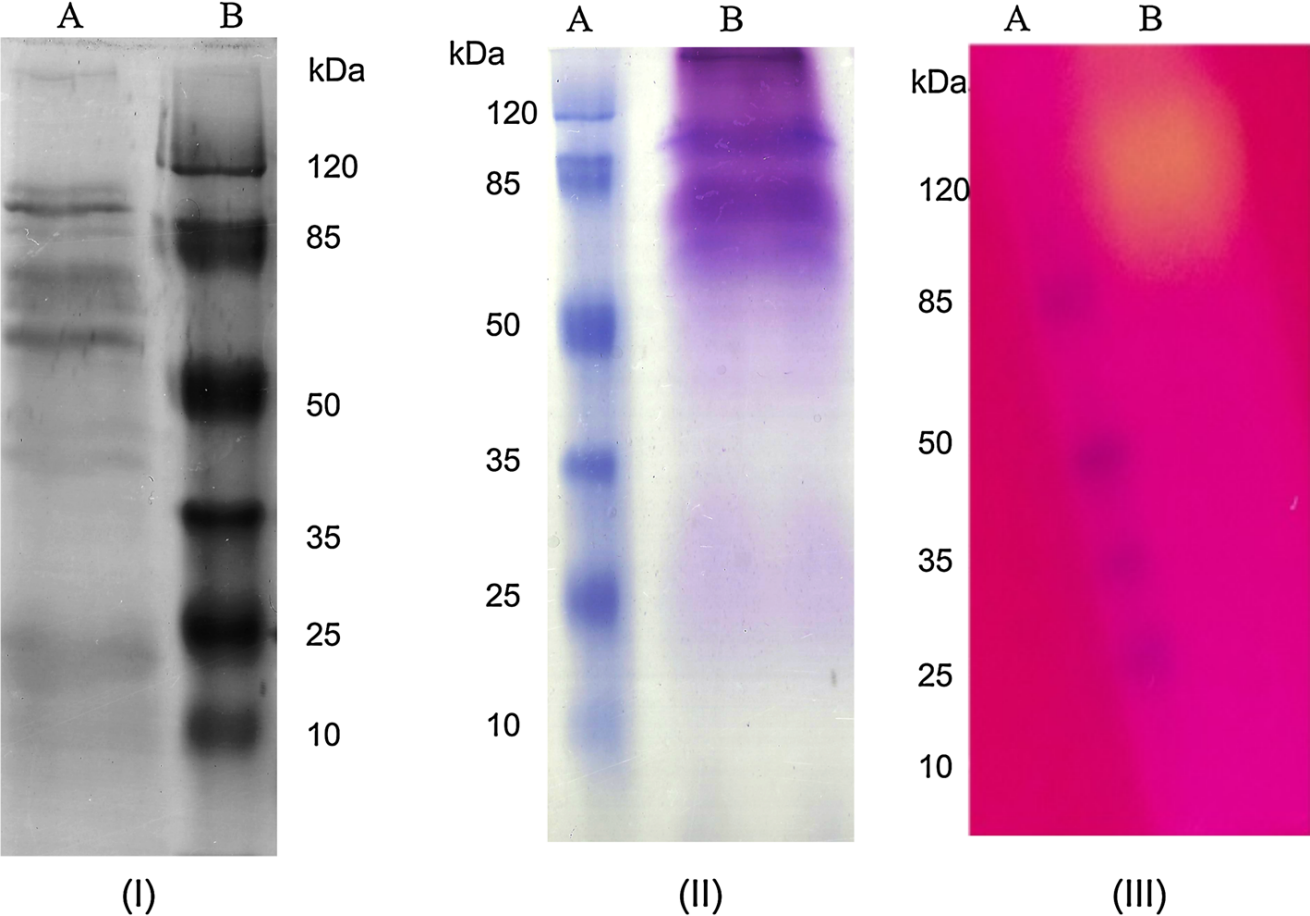
SDS-PAGE analysis: **I** Protein profile of crude supernatant obtained after growth of *M. spathulata* R25L270 for 120 h in liquid medium supplemented with olive oil. **II** Glycosylated protein profile of crude supernatant obtained after growth of *M. spathulata* R25L270 for 120 h in liquid medium supplemented with olive oil. **III** Zymographic analysis after SDS-NATIVE PAGE gel electrophoresis of crude supernatant obtained after growth of *M. spathulata* R25L270 for 120 h in liquid medium supplemented with olive oil.

**Figure 6:**
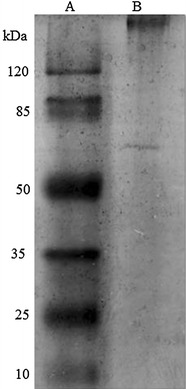
SDS-PAGE after purified lipases from *M. spathulata* R25L270. *Line A* molecular mass market. *Line B* purified lipase after phenyl-Sepharose immobilisation.

The profile of total and glycosylated proteins in the culture supernatant of *M. spathulata* R25L270 grown in liquid medium supplemented with olive oil (1% v/v) is shown in Figure 5. The presence of lipase was confirmed by enzymatic activity on PAGE (zymogram) using tributyrin as a substrate. The yellow band resulting from the release of fatty acids (Figure 5) confirms the presence of a lipase in the supernatant. The extracellular lipase was purified by hydrophobic interaction. First, a crude extract was obtained by growing the yeast *M. spathulata* R25L270 in liquid media enriched with macaúba cake (40 g L^−1^). The interfacial adsorption of lipases on hydrophobic supports at low ionic strength is a simple method for one-step purification and immobilization of these enzymes [11]. The purification/immobilization of lipases from *M. spathulata* R25L270 on phenyl-Sepharose support at 5 mM sodium phosphate buffer (pH 7) was carried out relatively rapidly, and almost 70% of the enzyme was adsorbed. The adsorbed enzymes were desorbed from the support by incubation in Triton X-100 solutions and the concentration of detergent required to fully desorb lipase activity was 0.09%. The amount of detergent required to release lipases from hydrophobic support depends on the hydrophobic and internal morphology of the support and the properties of each enzyme [37]. SDS-PAGE analysis of the desorbed proteins showed the presence of two bands, one corresponding to a molecular weight of >120 kDa and other corresponding to a molecular weight of >50 kDa, (Figure 6). These two proteins were subjected to analysis by mass spectrometry.

### Analysis by mass spectrometry

The two bands previously described were excised from the gel, subjected to tryptic digestion and analysed by RP-LC–MS/MS in an Easy-nLC II system coupled to an ion trap LTQ-Orbitrap-Velos-Pro mass spectrometer. With 20 peptide sequences and 11.40% putative protein coverage, protein 1 (>120 kDa) was identified as a protein from the transmembrane family MSFC (access: J6ET97) (Table 4). The second potential identification of protein 1 based on 9 peptide sequences and 17.86% putative protein coverage was a member of the lipase/esterase family (access: J6F4X8) (Table 4). Protein 2 (>50 kDa) was identified with 25 peptide sequences and 30.78% putative protein coverage as a member of the lipase-esterase family (access: J6F4X8); the other potential identity based on eight peptide sequences and 5.64% putative protein coverage was also a member of the protein transmembrane family MSFC (access: J6ET97) (Table 5). This finding suggests that both proteins are present in each band. The co-purification may have occurred due to the presence of hydrophobic regions on the surface of both proteins that bind to the support at low ionic strength.

**Table 4:** Peptides sequence of protein band 1 (>120 kDa)

A2	Sequence	Protein group accessions	Modifications	XCorr	Charge	MH+ [Da]	*m*/*z* [Da]
High	cQAADIVSSLNLQR	J6ET97	C1 (carbamidomethyl)	5.48	2	1,574.78203	787.89465
High	cPASDLTAALALSEKK	J6ET97	C1 (carbamidomethyl)	5.43	3	1,674.86588	558.96014
High	cPASDLTAALALSEK	J6ET97	C1 (carbamidomethyl)	5.14	2	1,546.77275	773.89001
High	FTNMASDcLQAK	J6ET97	C8 (carbamidomethyl)	4.76	2	1,385.61138	693.30933
High	SQDDTAcVcTDAYR	J6ET97	C7 (carbamidomethyl); C9 (carbamidomethyl)	4.74	2	1,661.63713	831.32220
High	cPSDITAALALQSK	J6ET97	C1 (carbamidomethyl)	4.65	2	1,474.75029	737.87878
High	CQAADIVSSLNLQR	J6ET97		4.14	2	1,517.76433	759.38580
High	FTNmASDcLQAK	J6ET97	M4 (oxidation); C8 (carbamidomethyl)	4.01	2	1,401.60527	701.30627
High	AGcSGPTDTGcLcTEK	J6ET97	C3 (carbamidomethyl); C11 (carbamidomethyl); C13 (carbamidomethyl)	4.00	2	1,713.67314	857.34021
High	LNFSTSTmGcLmK	J6ET97	M8 (oxidation); C10 (carbamidomethyl); M12 (oxidation)	3.90	2	1,521.66594	761.33661
High	LSPcALScVLDTLGK	J6ET97	C4 (carbamidomethyl); C8 (carbamidomethyl)	3.71	2	1,633.82121	817.41425
High	DLmDALALSSK	J6ET97	M3 (oxidation)	3.69	2	1,179.58745	590.29736
High	DLMDALALSSK	J6ET97		3.69	2	1,163.59197	582.29962
High	NNAAGcLTSNSK	J6ET97	C6 (carbamidomethyl)	3.65	2	1,236.55840	618.78284
High	cLIDAAAQLGcK	J6ET97	C1 (carbamidomethyl); C11 (carbamidomethyl)	3.59	2	1,319.64287	660.32507
High	LNFSTSTmGcLMK	J6ET97	M8 (oxidation); C10 (carbamidomethyl)	3.52	2	1,505.67363	753.34045
High	GSSDVCEATGIWR	J6ET97		3.48	2	1,380.61418	690.81073
High	cLVDAAATHGcK	J6ET97	C1 (carbamidomethyl); C11 (carbamidomethyl)	3.42	2	1,302.58611	651.79669
High	GSSDVcEATGIWR	J6ET97	C6 (carbamidomethyl)	3.24	2	1,437.63799	719.32263
High	LSPCALScVLDTLGK	J6ET97	C8 (carbamidomethyl)	2.96	2	1,576.79521	788.90125
High	LAALLGDAVFTLTR	J6F4X8		4.88	2	1,460.83855	730.92291
High	GAImNSGSmVPVDPVDGSR	J6F4X8	M4 (oxidation); M9 (oxidation)	4.85	2	1,920.86870	960.93799
High	SYGTGIEALNYGSPHR	J6F4X8		4.37	2	1,721.81670	861.41199
High	GAIMNSGSmVPVDPVDGSR	J6F4X8	M9 (oxidation)	4.21	2	1,904.87224	952.93976
High	SGLDVGKPFVFVAVNYR	J6F4X8		4.09	3	1,867.99619	623.33691
High	AEGIGNLGLLDQR	J6F4X8		3.54	2	1,355.71794	678.36261
Medium	TGIFNEIYPGFK	J6F4X8		3.44	2	1,385.69707	693.35217
High	ISEDcLTVNVIRPK	J6F4X8	C5 (carbamidomethyl)	3.11	2	1,643.87175	822.43951
High	GAIMNSGSMVPVDPVDGSR	J6F4X8		2.69	2	1,888.88420	944.94574

*J6ET97* [description: uncharacterized protein OS = *Trichosporon asahii* var. asahii (strain ATCC 90039/CBS 2479/JCM 2466/KCTC 7840/NCYC 2677/UAMH 7654) GN = A1Q1_03255 PE = 4 SV = 1 − (J6ET97_TRIAS)] and *J6F4X8* [uncharacterized protein OS = *Trichosporon asahii* var. asahii (strain ATCC 90039/CBS 2479/JCM 2466/KCTC 7840/NCYC 2677/UAMH 7654) GN = A1Q1_00392 PE = 4 SV = 1 − (J6F4X8_TRIAS)].

**Table 5:** Peptides sequence of protein 2 (>50 kDa)

A2	Sequence	Protein group accessions	Modifications	XCorr	Charge	MH+ [Da]	*m*/*z* [Da]
High	LAALLGDAVFTLTR	J6F4X8		5.12	2	1,460.84380	730.92554
High	PDGVFMTDSPDNLVSNKK	J6F4X8		4.94	3	1,963.93851	655.31769
High	SGLDVGKPFVFVAVNYR	J6F4X8		4.85	2	1,867.99834	934.50281
High	PDGVFmTDSPDNLVSNK	J6F4X8	M6 (oxidation)	4.81	2	1,851.83696	926.42212
High	GAImNSGSmVPVDPVDGSR	J6F4X8	M4 (oxidation); M9 (oxidation)	4.66	2	1,920.87639	960.94183
High	PDGVFMTDSPDNLVSNK	J6F4X8		4.58	2	1,835.84026	918.42377
High	PDGVFmTDSPDNLVSNKK	J6F4X8	M6 (oxidation)	4.53	3	1,979.92801	660.64752
High	SYGTGIEALNYGSPHR	J6F4X8		4.42	2	1,721.81645	861.41187
High	GAImNSGSMVPVDPVDGSR	J6F4X8	M4 (oxidation)	4.30	2	1,904.87676	952.94202
High	ISEDCLTVNVIRPK	J6F4X8		4.14	2	1,586.83379	793.92053
High	AEGIGNLGLLDQR	J6F4X8		4.14	2	1,355.72612	678.36670
High	TGIFNEIYPGFK	J6F4X8		3.80	2	1,385.70098	693.35413
High	ISEDcLTVNVIRPK	J6F4X8	C5 (carbamidomethyl)	3.73	2	1,643.87407	822.44067
High	GAIMNSGSMVPVDPVDGSR	J6F4X8		3.67	2	1,888.88225	944.94476
Medium	AGcDTAPDSLQcLR	J6F4X8	C3 (carbamidomethyl); C12 (carbamidomethyl)	3.50	2	1,563.68144	782.34436
Medium	ISEDcLTVNVIR	J6F4X8	C5 (carbamidomethyl)	3.49	2	1,418.72783	709.86755
High	KAGcDTAPDSLQcLR	J6F4X8	C4 (carbamidomethyl); C13 (carbamidomethyl)	3.41	3	1,691.77201	564.59552
High	KDPLVNWDATK	J6F4X8		3.33	2	1,286.66814	643.83771
High	QLINFYAmYFK	J6F4X8	M8 (oxidation)	3.32	2	1,453.71562	727.36145
High	ISEDCLTVNVIR	J6F4X8		3.29	2	1,361.70427	681.35577
High	PFVFVAVNYR	J6F4X8; N4V6R7		3.28	2	1,211.65520	606.33124
Medium	QLINFYAMYFK	J6F4X8		3.17	2	1,437.72014	719.36371
Medium	TAYGALGLRL	J6F4X8		3.11	2	1,034.59783	517.80255
Medium	VAGWGFMPGK	J6F4X8		3.07	2	1,049.52117	525.26422
High	TGIFNEIYPGFKR	J6F4X8		2.89	3	1,541.80661	514.60706
High	cPASDLTAALALSEK	J6ET97	C1 (carbamidomethyl)	4.46	2	1,546.77397	773.89063
High	cQAADIVSSLNLQR	J6ET97	C1 (carbamidomethyl)	4.23	2	1,574.78728	787.89728
High	FTNMASDcLQAK	J6ET97	C8 (carbamidomethyl)	4.18	2	1,385.61431	693.31079
High	DLmDALALSSK	J6ET97	M3 (oxidation)	3.80	2	1,179.59075	590.29901
High	NNAAGcLTSNSK	J6ET97	C6 (carbamidomethyl)	3.72	2	1,236.55889	618.78308
High	cPSDITAALALQSK	J6ET97	C1 (carbamidomethyl)	3.70	2	1,474.75408	737.88068
High	DLMDALALSSK	J6ET97		3.56	2	1,163.59526	582.30127
Medium	cPASDLTAALALSEKK	J6ET97	C1 (carbamidomethyl)	3.06	3	1,674.86753	558.96069

*J6ET97* [description: uncharacterized protein OS = *Trichosporon asahii* var. asahii (strain ATCC 90039/CBS 2479/JCM 2466/KCTC 7840/NCYC 2677/UAMH 7654) GN = A1Q1_03255 PE = 4 SV = 1 − (J6ET97_TRIAS)] and *J6F4X8* [uncharacterized protein OS = *Trichosporon asahii* var. asahii (strain ATCC 90039/CBS 2479/JCM 2466/KCTC 7840/NCYC 2677/UAMH 7654) GN = A1Q1_00392 PE = 4 SV = 1 − (J6F4X8_TRIAS)].

### Application of lipases to catalyse vegetable and fish oil hydrolysis

The inclusion of enzymatic processes to oil modification in the oleochemical industry is promising and leads to improved environmental conditions, namely the lowest possible power consumption and waste generation. It also allows the production of unsaturated fatty acids without oxidation [7]. Fatty acids are currently produced by extreme conditions of temperature and pressure that promote the polymerisation of fats and formation of by-products that need to be removed by purification and distillation steps [7]. In this context, the potential application of lipases from *M. spathulata* R25L270 for oil hydrolysis was determined using different vegetable oils emulsified with gum arabic solution. Table 6 shows that the enzyme was able to produce free fatty acids from all oils tested and preferentially catalysed sesame, olive and sunflower oils, showing enzymatic activity values of 46.86, 43.26 and 45.66 U mg^−1^, respectively.

**Table 6:** Hydrolyzes of commercial oils by lipase from *M.* *spathulata* R25L270

Vegetal oil	Fatty acid (µmoles)	Hydrolysis activity (U mg^−1^)
Corn	46.50 ± 9.30	36.05 ± 7.21
Soya	52.70 ± 5.37	33.64 ± 4.16
Sesame	116.25 ± 6.57	46.86 ± 5.10
Almond	55.80 ± 9.30	36.05 ± 7.21
Olive	58.90 ± 10.74	43.26 ± 0.0
Canola	46.50 ± 9.30	36.05 ± 7.21
Sunflower	58.90 ± 5.37	45.66 ± 4.16

The lipase from *M. spathulata* R25L270 exhibits interesting catalytic properties with regard to selectivity. Some lipases present specificity regarding the type of fatty acid and degree of saturation. Lipase selectivity depends on the enzyme form (e.g., free or immobilised), enzyme source (e.g., plant, animal, microbial), and reaction conditions (e.g., pH, solvents and temperature). These conditions alter the conformation and flexibility of the protein. The enzyme efficiently catalysed the selective hydrolysis of sardine oil. The release of omega-3 fatty acids [e.g., eicosapentaenoic acid (EPA) and docosahexaenoic acid (DHA)] from fish oil represents the first key step in the preparation of highly enriched triglyceride products, which have been described as excellent functional ingredients [18]. As shown in Table 7, the release rate of EPA was approximately fivefold higher than the release of DHA, especially during the first hour of the reaction. The EPA/DHA selectivity of this new extracellular lipase could make it attractive for industrial application, e.g., allowing the production of 5:1 EPA:DHA mixtures from sardine oil hydrolysis. Based on the results obtained in the present study, we speculate that the lipase from *M. spathulata* R25L270, which showed potential for degrading different oils and substrate selectivity, has potential for application in the oleochemical industry and also in oil-laden effluent treatment.

**Table 7:** Hydrolysis of sardine oil catalyzed by *M.* *spathulata* R25L270 lipase and ratio between the releases of EPA versus the release of DHA during the time

Time (h)	EPA (µM)	DHA (µM)	Ratio (EPA/DHA)
24	17.68	3.56	4.97
48	20.93	5.24	3.99
72	37.01	11.94	3.10
96	45.20	14.31	3.16
120	54.23	19.34	2.80

*EPA* eicosapentaenoic acid, *DHA* docosahexaenoic acid

## Conclusions

A mixture of macaúba cake and residual oil instead of olive oil was successfully used for induction of lipase secretion by *M. spathulata* R25L270. The lipase functionality in a wide temperature and pH range revealed its potential for utilisation in bioprocesses. The enzyme efficiently hydrolysed different oils and showed selectivity for generating EPA from fish oil. This is the first study demonstrating the use of macaúba cake as a substrate for lipase production and the application of lipases from *M. spathulata* in oil hydrolysis. Lipase immobilisation is a strategy to explore the lipolytic potential of this new lipase source.

## Abbreviations

MC: macaúba cake
RO: residual oil
EPA: eicosapentaenoic acid
GYMP broth: glucose, yeast extract, malt extract, NaH_2_PO_4_ and glycerol
*p*-NPP: *p*-nitrophenyl palmitate
*p*NP: *p*-nitrophenol
SDS-PAGE: sodium dodecyl sulfate polyacrylamide gel electrophoresis
MS: mass spectrometry
ACN: acetonitriler
RP-LCMS/MS: reverse phase liquid chromatography mass spectrometry in tandem
FDR: false discovery rate
LA: supernatant lipase activity
LP: supernatant total protein
